# Supplementary figures and images for: In-Depth Analysis of Bacillus anthracis 16S rRNA Genes and Transcripts Reveals Intra- and Intergenomic Diversity and Facilitates Anthrax Detection
Source: mSystems. 2022 Jan 25;7(1):e01361-21. doi: 10.1128/msystems.01361-21 (PMC8788319; doi:10.1128/msystems.01361-21)

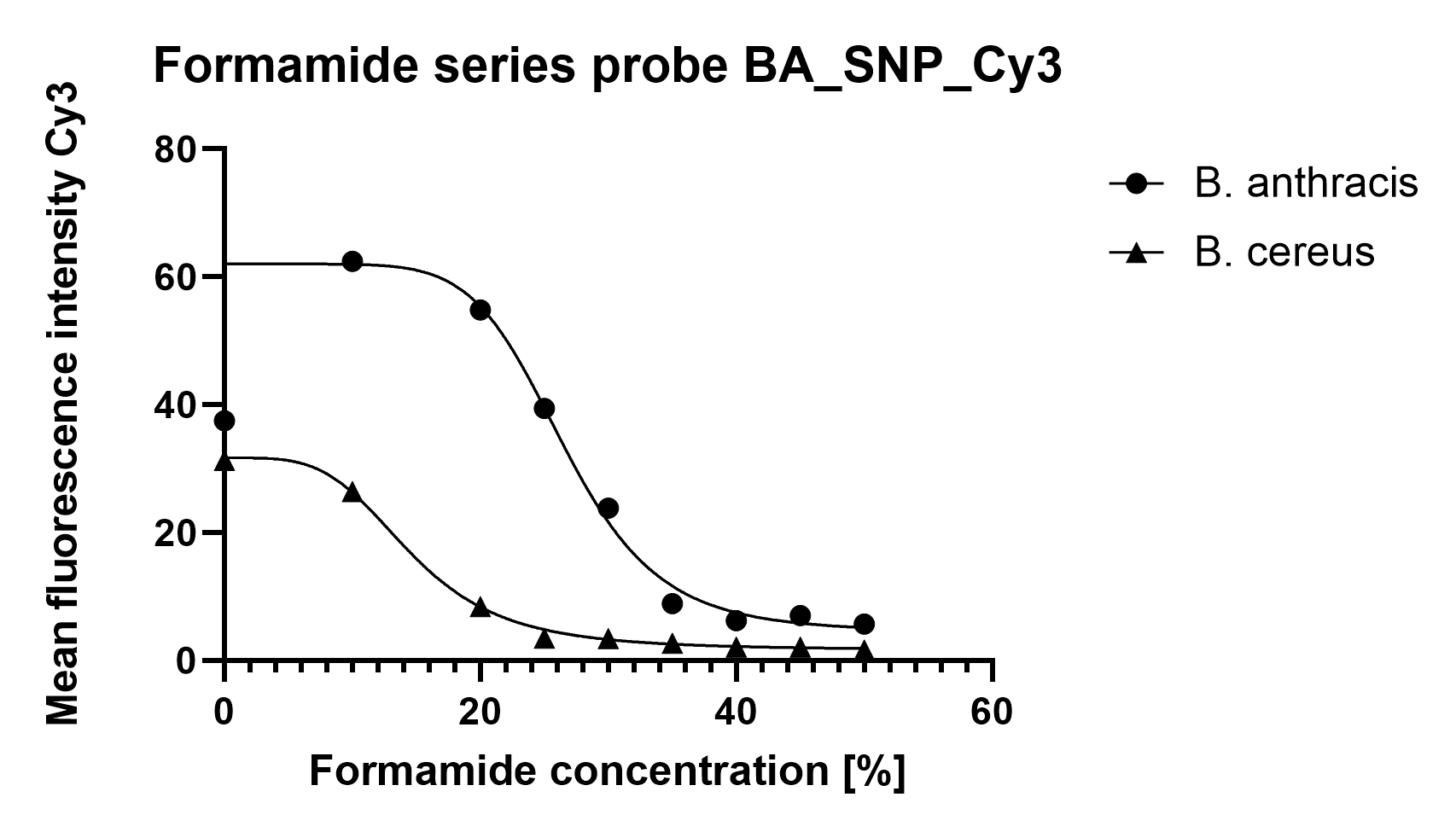

Supplement: FIG S1 [file msystems.01361-21-sf001.tif]

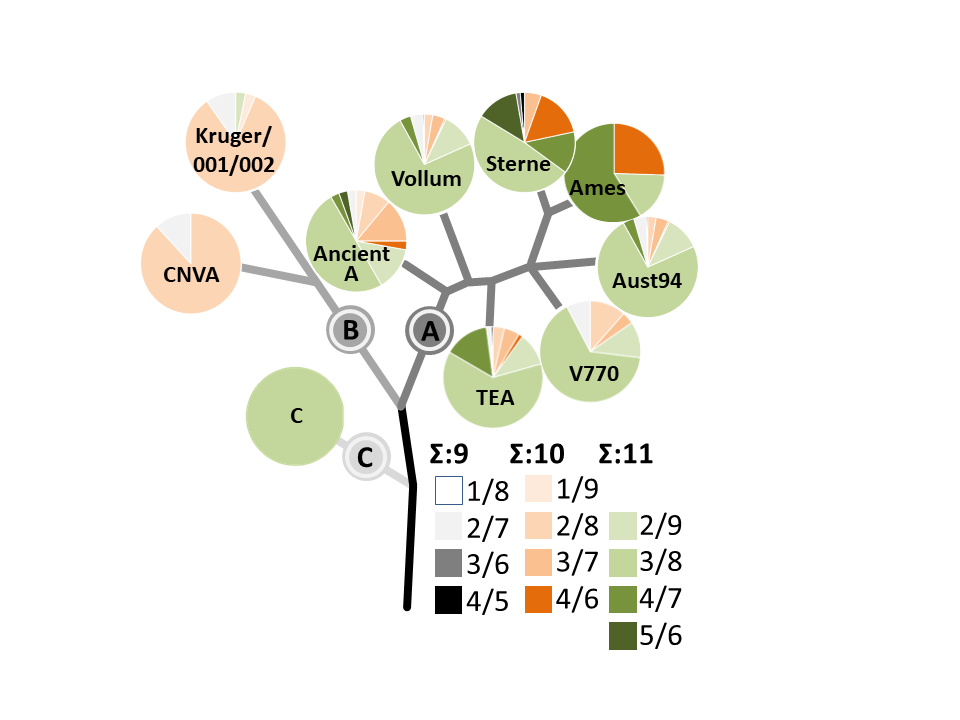

Supplement: FIG S2 [file msystems.01361-21-sf002.tif]
